# Cross-sectional analysis of self-efficacy and social capital in a community-based healthy village project in Santa Cruz, Bolivia

**DOI:** 10.1186/s12914-015-0054-y

**Published:** 2015-06-20

**Authors:** Motoyuki Yuasa, Yoshihisa Shirayama, Keiichi Osato, Cesar Miranda, Julia Condore, Roxana Siles

**Affiliations:** Department of Public Health, Juntendo University Graduate School of Medicine, Bunkyo-ku, Tokyo Japan; Bolivia Office, Japan International Cooperation Agency, LaPaz, Bolivia; Health Promotion Unit, Government Health Office, Santa Cruz, Bolivia

**Keywords:** Health promotion, Self-efficacy, Social capital, Bolivia

## Abstract

**Background:**

An assessment of self-efficacy and social capital may have the potential to detect an effect of dynamic, complex and comprehensive collective actions in community-based health promotion. In 2003, a healthy village project was launched in Santa Cruz, Bolivia with technical assistance from the Japan International Cooperation Agency (JICA). The originally developed FORSA (*Fortalecimiento de Redes de Salud*) model accounted for participatory processes in which people could improve their health and well-being through individual behavioral changes and family/community-driven activities. This study aimed to examine the extent of self-efficacy and social capital obtained via project activities by a cross-sectional analysis.

**Methods:**

We randomly selected 340 subjects from the healthy village project site and 113 subjects from a control area. Both groups were interviewed using the same structured questionnaire. Self-efficacy was assessed with a General Self-Efficacy Scale (GSES), while social capital was measured as the frequency of formal group participation in community meetings during the past three months, perceived social solidarity, and general trust.

**Results:**

The study results showed that the participants in the project site had higher self-efficacy and social capital compared to those in the control site. The number of times a subject participated in the health committee activities was positively associated with the self-efficacy scale. Regarding social capital, females and lower-educated people were more likely to have had more frequent participation in formal groups; males and higher-educated participants showed less formal group participation, but more generosity to contribute money for the community. The main perceived benefit of participation in formal group activities varied among individuals.

**Conclusion:**

The findings suggest that people in the healthy village project site have higher self-efficacy, especially those with active participation in the health committee activities. To recruit more participants in future healthy village projects, we should consider the gender and level of education, and match the perceived benefits of participants accordingly.

## Background

Community-based health promotion programs are widely implemented worldwide [1]. Particularly in deprived rural communities in less-developed countries, such programs have been adopted in a community development approach for the improvement of health. Bolivia, one of the lowest income countries in Latin America, has participated in health-related projects for comprehensive community development [2, 3]. For more than three decades, the Japan International Cooperation Agency (JICA) has provided Bolivia with official aid focusing on the development of health professionals’ capacity and the establishment of a physical infrastructure. In 2003, the JICA launched a healthy village project in the province of Santa Cruz in Bolivia. This project developed the so-called FORSA (*Fortalecimiento de Redes de Salud*) model, a simplified version of the PRECEDE-PROCEED model [4], and has technically supported the implementation of community-based health promotion using this model.

The model accounted for participatory processes in which people could improve health and well-being through individual behavioral changes and family/community-driven activities [5]. Trained health professionals, such as doctors and registered nurses, were in charge of community engagement using the model. The stepwise process of the FORSA model enabled people in the community to ensure their collective aim, determine primary behaviors for achieving the aim, identify three factors to facilitate targeted behaviors to change, and consequently create a health promotion plan pertinent to addressing those three factors. In identifying the three antecedents and reinforcing factors to initiate and sustain the behavioral change, the people in the community classified predisposing factors. These factors included knowledge, attitude, belief and skills, the reinforcing factor of neighborhood support, and the enabling factor of availability of a health professional’s support. Subsequently, the participants developed their own health promotion plan. We hypothesized that assessment of self-efficacy and social capital might have the potential to detect an effect of dynamic, complex, and comprehensive collective actions in community-based health promotion based on our experience [6]. Thus, for the purpose of evaluating this healthy village project, we examined the extent of self-efficacy and social capital among the participants in the project.

Self-efficacy is widely used as a cognitive variable to assess human capital. Self-efficacy is defined as a conviction that one can successfully execute behavior required to produce outcome [7]. Self-efficacy depends on what a particular behavior is in different settings, e.g., quitting smoking or starting physical activity. However, the FORSA model approach addressed the development of self-efficacy as a whole, not for specific situations. Therefore, the General Self-Efficacy Scale (GSES) was used to assess the development of human capital in the project [8].

Social capital refers to the quantity and quality of social relationships, such as formal and informal social connections, as well as norms of reciprocity and trust that exist within the community [9]. Although the construct of social capital is recognized to induce a potential negative consequence [10], social capital is generally endorsed to play a crucial role for transformative social engagement devoted to promoting health [11, 12]. In the context of Latin America, concern has been growing about social capital in health promotion [13]. In the FORSA model project, the community residents were encouraged to mobilize and autonomously engage in the collective action. The participants were thus expected to increase social solidarity thorough their participation.

This study aimed to examine the extent of self-efficacy and social capital in the healthy village project site compared to people in a non-project control site, in Santa Cruz, Bolivia.

## Methods

### Study participants

During January and February 2012, we recruited study participants with a two-stage cluster sampling method. We numbered all clusters of communities in sixteen project municipalities and twelve control areas located in the fourth region of the health network in the province of Santa Cruz. Reference subjects were recruited from the control municipalities where the healthy village project had never been implemented. We used a table of random digits to select 29 communities in the project municipalities and 14 communities in the control municipalities. In the project communities, the study subjects were randomly selected by systematic extraction from a list of households the project provided. In the control group, we had no project and had no list of households, so instead selected houses randomly using an area map drawn by the personnel of the public health centers and posts. Each of the residents dwelling at the chosen house was recruited. We collected the self-administered consent form from 340 participants in the project site and 113 in the control site. Local college students who had been engaged in a public health specialty course interviewed the participants in both sites using a structured questionnaire.

The present study was carried out as part of the healthy village project which the provincial government of Santa Cruz, Bolivia administered. The protocol was also approved by the Ethics Committee for Epidemiological Studies of Juntendo University Graduate School of Medicine in Japan. The study was carefully conducted in accordance with the declaration of Helsinki.

### Measurement variables

A cross-sectional survey was carried out to examine variables regarding GSES and social capital in the project and control communities. We measured the frequency of formal group participation in community meetings, perceived social solidarity, and general trust as social capital indicators.

GSES is a validated scale of general self-efficacy that includes 10 questions scored on a four-point Likert scale [14]. The scale ranges from 10 to 40, with a higher score indicating stronger general self-efficacy. The concept of formal group participation in community meetings as structural social capital and perceived social solidarity as cognitive social capital were adopted from the relevant questions in the Integrated Questionnaire developed by the World Bank [15]. Formal group participation was measured as the frequency of participation in formal group meetings in the past three months. A higher number for formal group participation indicates a stronger connection between the participants and the community activities. Perceived social solidarity was measured by asking the question, “In general, do you agree or disagree: most people in this village are willing to help if you need it?” ”Strongly Agree” was given a higher score, which meant a higher perceived social solidarity. Perceived social solidarity was also measured financially. It was determined by the questions, “If a community project does not directly benefit you but has benefits for many others in the village, would you contribute time?” and “If a community project does not directly benefit you but has benefits for many others in the village, would you contribute money?” each with a dichotomous response: yes or no. An answer of “yes” was coded as one and an answer of “no” was coded as zero. General trust was defined as the extent to which one believes that others will not act to exploit one’s vulnerabilities [16] or as the default expectation of other people’s trustworthiness [17]. It was assessed on a five-point Likert scale by asking the question, “Generally speaking, would you say that most people can be trusted?” A higher score means a higher perceived general trust. Finally, we asked the study participants what they felt was the main benefit of joining formal group activities, including health promoting activities.

### Analyses

Characteristics of the participants included age (years), gender (male or female), level of education (none, primary school, secondary school, vocational school, or university/college), and how many times they participated in formal group activities including health promoting activities. We analyzed the data by a chi-square test, t-test, or one way analysis of variance, and with non-parametric methods of the Mann–Whitney test or the Kruskal-Wallis test. The Cronbach’s alpha was calculated to report the reliability of the GSES results.

Analyses were conducted on the overall data, and with stratification by gender and by three educational levels (low level classified as none and primary school; middle level as secondary school; and high level as vocational school or university/college). Statistical analyses were performed using IBM SPSS statistics 21 (IBM Inc., USA). Results were considered statistically significant if the *p* value was less than 0.05.

## Results

### Characteristics of the study subjects

Table 1 shows the characteristics of a total of 453 study participants with a mean age of 32.8 (SD = 10.0). There were 111 males and 342 females. Age did not differ significantly between the project site and the control site. However, the number of females and the educational level in the project site were statistically higher than those in the control site, despite random sampling. In the project site, 51 subjects (15.0 %) did not participate in formal group activities, but 289 (85.0 %) participated at least once. In the control site, 95 (84.1 %) did not participate in such activities.

**Table 1:** Characteristics of the subjects

	Project site (n = 340)	Control site (n = 113)	*p*-value
Age (years)			0.559*
mean	32.6 ± 9.2	33.3 ± 12.0	
minimum/maximum	17 / 73	16 / 73	
Gender, n (%)			<0.001^#^
Male	63 (18.5)	48 (42.5)	
Female	277 (81.5)	65 (57.5)	
Educational attainment, n (%)			0.021^#^
None	6 (1.8)	8 (7.1)	
Primary school	74 (21.8)	31 (27.4)	
Secondary school	184 (54.1)	54 (47.8)	
Vocational school	31 (9.1)	11 (9.7)	
University/college	45 (13.2)	9 (8.0)	
Number of times participated in formal group activities including health promoting activities, n (%)			
0	51 (15.0)	95 (84.1)	
1	39 (11.5)	12 (10.6)	
2	37 (10.9)	1 (0.9)	
3	78 (22.9)	1 (0.9)	
4	56 (16.5)	1 (0.9)	
5	13 (3.8)	1 (0.9)	
6	23 (6.8)	0 (0.0)	
7	13 (3.8)	0 (0.0)	
8	16 (4.7)	2 (1.8)	
9	12 (3.5)	0 (0.0)	
10 times or more	2 (0.6)	0 (0.0)	

*t-test

^#^chi-square test

### Self-efficacy and social capital

Table 2 shows the results with overall data in which the GSES in the project site was significantly higher than that in control site. The Cronbach’s alpha of the GSES (10 questions) was 0.88. With regard to the scores of social capital, such as formal group participation, perceived solidarity and general trust, the project participants had significantly stronger social capital than the control site. The participants in the project site were more likely to respond that they contributed their time for the benefit of the community, irrespective of whether they received a benefit, than those in the control site. However, no significant difference in financial contributions was observed among the subjects in the project site and the control site.

**Table 2:** Self-efficacy and social capital among the subjects

	Project site (n = 340)	Control site (n = 113)	*p*-value
General self-efficacy (mean)	33.5 ± 4.5	29.6 ± 5.7	<0.001*
Social capital (median)			
Formal group participation	3	0	<0.001^#^
Perceived social solidarity	4	4	<0.001^#^
Perceived solidarity in time	1	1	<0.001^#^
Perceived solidarity in money	0	0	0.349^#^
General trust	4	3	<0.001^#^

*t-test

^#^Mann–Whitney test

**Fig. 1:**
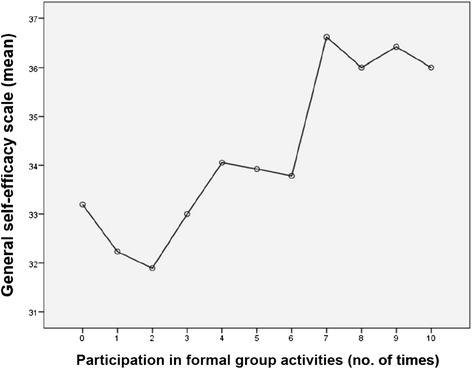
Self-efficacy and participation in formal group activities in the project site

Focusing on the project subjects, GSES was positively associated with the number of times participated in formal group activities (Fig. 1). For the results stratified analysis by gender, females participated in formal group meetings more frequently than males; in contrast, males were more likely than females to answer that they would contribute money (Table 3). As shown in Table 4, when classified by three educational levels, higher-educated participants showed a higher GSES and were more likely to answer that they would contribute money for the community, though they had less participation in formal group meetings.

**Table 3:** Self-efficacy and social capital by gender in the project site

	Males (n = 63)	Females (n = 277)	*p*-value
General self-efficacy (mean)	34.0 ± 4.4	33.4 ± 4.5	0.301*
Social capital (median)			
Formal group participation	1	3	<0.001^#^
Perceived social solidarity	5	4	0.117^#^
Perceived solidarity in time	1	1	0.237^#^
Perceived solidarity in money	1	0	0.001^#^
General trust	4	4	0.240^#^

*t-test

^#^chi-square test

**Table 4:** Self-efficacy and social capital by educational level in the project site

	Low^a^ (n = 80)	Middle^a^ (n = 184)	High^a^ (n = 76)	*p*-value
General self-efficacy (mean)	33.0 ± 4.6	33.2 ± 4.5	34.9 ± 4.3	0.011*
Social capital (median)				
Formal group participation	4	3	3	0.003^#^
Perceived social solidarity	5	4	5	0.394^#^
Perceived solidarity in time	1	1	1	0.166^#^
Perceived solidarity in money	0	0	1	0.002^#^
General trust	4	4	4	0.050^#^

^a^Low, none and primary school middle, secondary school; high level, vocational school or university/college.

*one-way analysis of variance

^#^Kruskal-Wallis test

### Motivation of participation in group activities

Table 5 summarizes the reasons why the participants joined in formal group activities. Almost half of the respondents joined to offer benefits for their community. They also participated for their current and future advantages. There were few who joined for enjoyment.

**Table 5:** Reasons for participation in formal group activities (project and control sites combined)

Responses explaining the main benefit of participation	n (%)
Improves my household’s current livelihood or access to services	61 (13.5)
Important in times of emergency/in future	73 (16.1)
Benefits the community	239 (52.8)
Enjoyment/recreation	8 (1.8)
Spiritual, social status, self-esteem	5 (1.1)
Other	67 (14.8)

## Discussion

The current cross-sectional study reported that people in the project site participated more frequently in formal group activities including health promoting activities. This coincided with better self-efficacy and social capital compared with those in the non-project site.

The study area, Santa Cruz in Bolivia, has strong cultural influences from Brazil, because of geography and history. In Brazil, social capital is known as one of the lowest internationally. Likewise, low social capital was expected in Santa Cruz, though it was never studied before. However, in the project site, the social capital was reported higher compared with that in the control site. The social capital can be improved simply by establishing a community group. The FORSA project, which aimed to enhance communication between the community and health staff, might have contributed to the improved self-efficacy and social capital.

Previous studies also observed higher GSES of participants in community development and health promoting activities [18]. People with high/moderate self-efficacy at baseline were more likely to join additional health promotion activities compared to those with low self-efficacy [19, 20], while health promotion intervention could significantly enhance the self-efficacy of participants and vice versa [21, 22]. Our study was a cross-sectional analysis, so we cannot determine the causation, but found a positive association between participation and GSES anyway. It should be noted that Gatewood et al. reported no significant association of self-efficacy with health promotion activities when adjusted for three levels of program participation, such as full, minimum and none [23]. Not only the frequency of participation, but also quality of participation should be studied in our future research.

We found better structural social capital in the project site compared with the non-project site, which was indicated by the frequency variable of formal group participation. The primary purpose of the healthy village project was to encourage the residents to participate in community meetings and improve their health-related issues together. The study showed females and lower-educated people were more likely to attended community meetings in the project site (Tables 3 and 4). It seems common that females are usually more motivated and empowered through a community development program [24].

Many previous researchers explained that a community-based health promotion intervention advanced a cognitive form of social capital and subsequently generated collective actions to affect health [25, 26]. In our study, likewise, significantly higher perceived solidarity and general trust among the project participants suggested that health promotion intervention might be associated with an enhanced form of cognitive social capital. Furthermore, we observed that the participants in the project site were willing to contribute their time to the community, even if there was no direct benefit to themselves. Meanwhile, we saw no significant difference in perceived solidarity in terms of money (Table 2), but males or higher-educated participants in the project site appeared to be more likely to contribute money for the public (Tables 3 and 4). The results seem to be realistic due to both the project area and the control area being economically deprived, and males [27] and higher-educated people [28] were found to be more generous with their own money than females and lower-educated people.

In general, primary health care professionals are less likely to have more concern toward a community development approach than a medical approach [29]. Lack of health personnel’s interest in community development may be due to poor instruction for involving communities in health activities, a paucity of leadership, and the considerable difficulty of visualizing the outcomes of their activities [30, 31]. Considering these factors, the project has thus far coached health professionals for mobilization of the community, and the current research findings should motivate professionals to promote the FORSA model activity. In fact, this model has recently been scaled up to other provinces in the nation.

This study has some limitations. First, as the research was a cross-sectional design, we could not prove that the project was directly related with enhancement of self-efficacy and social capital. We need a further longitudinal study to elucidate whether the participatory approach of the FORSA model caused an increase in self-efficacy and social capital. Second, regardless of random sampling, we found a gender deviance (more females) and higher educational attainment of the participants in the project site compared to those in the control site. Third, the research questionnaire was developed with the priority of being brief and easy to complete, which resulted in insufficient data inclusion on related confounding factors. It is assumed that other possible confounders, e.g., income, level and quality of participation, and social status, may affect self-efficacy and social capital.

## Conclusion

We hypothesized that self-efficacy and social capital may be good parameters to study the effects of community-based health promotion activities. Our study revealed that the project participants appeared to have higher self-efficacy and social capital compared with individuals from the non-project area. In particular, females and lower-educated persons were more likely to participate in formal group meetings; in contrast, males and higher-educated people showed less formal group participation and less hesitation to contribute money for the public benefit. To recruit more participants in future healthy village projects, we should consider the gender and level of education, and match the perceived benefits of participants accordingly.

## References

[1] Li IC (2004). The effectiveness of a health promotion program for the low-income elderly in Taipei. Taiwan J Community Health.

[2] Eder C, Schooley J, Fullerton J, Murguia J (2012). Assessing impact and sustainability of health, water, and sanitation interventions in Bolivia six years post-project. Rev Panam Salud Publica.

[3] Velasquez J, Knatterud-Hubinger N, Narr D, Mendenhall T, Solheim C (2011). Mano a Mano: Improving health in impoverished Bolivian communities through community-based participatory research. Fam Syst Health.

[4] Green WL, Kreuter WM (1999). Health promotion planning; 3rd edition: An educational and ecological approach.

[5] Kobeissi L, Nakkash R, Ghantous Z, Saad MA, Yassin N (2011). Evaluating a community based participatory approach to research with disadvantaged women in the southern suburbs of Beirut. J Community Health.

[6] Yuasa M, de Sa RF, Pincovsky S, Shimanouchi N (2007). Emergence Model of social and human capital and its application to the Healthy Municipalities project in Northeast Brazil. Health Promot Int.

[7] Bandura A (1977). Self-efficacy: toward a unifying theory of behavioral change. Psychol Rev.

[8] Bosscher RJ, Smit JH (1998). Confirmatory factor analysis of the General Self-Efficacy Scale. Behav Res Ther.

[9] Kawachi I, Berkman L, Berkman L, Kawachi I (2000). Social cohesion, social capital, and health. Social epidemiology.

[10] Wakefield SE, Poland B (2005). Family, friend or foe? Critical reflections on the relevance and role of social capital in health promotion and community development. Soc Sci Med.

[11] Griffiths R, Horsfall J, Moore M, Lane D, Kroon V, Langdon R (2007). Assessment of health, well-being and social connections: a survey of women living in Western Sydney. Int J Nurs Pract.

[12] Murayama H, Wakui T, Arami R, Sugawara I, Yoshie S (2012). Contextual effect of different components of social capital on health in a suburban city of the greater Tokyo area: a multilevel analysis. Soc Sci Med.

[13] Sapag JC, Kawachi I (2007). [Social capital and health promotion in Latin America]. Rev. Saude Publica.

[14] Luszczynska A, Scholz U, Schwarzer R (2005). The general self-efficacy scale: multicultural validation studies. J Psychol.

[15] Grootaert C, Narayan D, Nyhan Jones V, Woolcock M. (2004). Measuring social capital; An Integrated Questionnaire. The World Bank, retrieved March 20, 2013, from https://openknowledge.worldbank.org/bitstream/handle/10986/15033/281100PAPER0Measuring0social0capital.pdf?sequence=1.

[16] Mayer RC, Davis JH, Schoorman FD (1995). An Integrative Model of Organizational Trust. Acad. Manage. Rev.

[17] Yamagishi T, Kikuchi M (1999). Trust, gullibility, and social intelligence. Asian J Soc Psychol.

[18] Kwong EW, Kwan AY (2007). Participation in health-promoting behaviour: influences on community-dwelling older Chinese people. J Adv Nurs.

[19] Kaiser BL, Brown RL, Baumann LC (2010). Perceived influences on physical activity and diet in low-income adults from two rural counties. Nurs Res.

[20] Tayama J, Yamasaki H, Tamai M (2012). Effect of baseline self-efficacy on physical activity and psychological stress after a one-week pedometer intervention. Percept Mot Skills.

[21] Dutton GR, Tan F, Provost BC, Sorenson JL, Allen B, Smith D (2009). Relationship between self-efficacy and physical activity among patients with type 2 diabetes. J Behav Med.

[22] Wu MP, Wu SF, Wang TC, Kao MJ, Yang WL (2012). Effectiveness of a community-based health promotion program targeting people with hypertension and high cholesterol. Nursing & health sciences.

[23] Gatewood JG, Litchfield RE, Ryan SJ, Geadelmann JD, Pendergast JF, Ullom KK (2008). Perceived barriers to community-based health promotion program participation. Am J Health Behav.

[24] Rios R, Olmedo C, Fernandez L (2007). Empowered women from rural areas of Bolivia promote community development. Promot Educ.

[25] Linden-Bostrom M, Persson C, Eriksson C (2010). Neighbourhood characteristics, social capital and self-rated health–a population-based survey in Sweden. BMC Public Health.

[26] Shan H, Muhajarine N, Loptson K, Jeffery B (2014). Building social capital as a pathway to success: community development practices of an early childhood intervention program in Canada. Health Promot Int.

[27] Bonevski B, Bryant J, Lynagh M, Paul C (2012). Money as motivation to quit: a survey of a non-random Australian sample of socially disadvantaged smokers' views of the acceptability of cash incentives. Prev Med.

[28] Yuasa M, Ukawa S, Ikeno T (2012). Relationship of general trust with individual related factors among frail elderly residents at home in Hokkaido rural areas in Japan. Health (N Y).

[29] Hogg R, Hanley J (2008). Community development in primary care: opportunities and challenges. Community Pract.

[30] Nutbeam D, Smith C, Murphy S, Catford J (1993). Maintaining evaluation designs in long term community based health promotion programmes: Heartbeat Wales case study. J Epidemiol Community Health.

[31] Smillie C (1992). Preparing health professionals for a collaborative health promotion role. Can J Public Health.

